# Analgesic Efficacy of COX-2 Inhibitors in Periodontal Surgery: A Systematic Review and Meta-Analysis

**DOI:** 10.3390/healthcare11071054

**Published:** 2023-04-06

**Authors:** Mario Alberto Isiordia-Espinoza, Eduardo Gómez-Sánchez, Itzel Joselyn Mora-Falcón, Iván Agustín Amador-Beas, Adriana Hernández-Gómez, Nicolás Addiel Serafín-Higuera, Lorenzo Franco-de la Torre

**Affiliations:** 1 Instituto de Investigación en Ciencias Médicas, Departamento de Clínicas, División de Ciencias Biomédicas, Centro Universitario de los Altos, Universidad de Guadalajara, Av. Rafael Casillas Aceves No. 1200, Tepatitlán de Morelos 47620, Jalisco, Mexico; 2 División de Disciplinas Clínicas, Centro Universitario de Ciencias de la Salud, Universidad de Guadalajara, Sierra Mojada 950, Colonia Independencia Oriente, Guadalajara 44340, Mexico; 3 Centro de Ciencias de la Salud, Facultad de Odontología, Universidad Autónoma de Baja California, Campus Mexicali, Mexicali 21040, Mexico

**Keywords:** analgesic efficacy, COX-2 inhibitors, glucocorticoids, non-steroidal anti-inflammatory analgesics, periodontal surgery

## Abstract

The objective of this systematic review and meta-analysis was to evaluate the analgesic efficacy of COX-2 inhibitors versus other drugs in periodontal surgery. Two researchers searched PubMed, Google Scholar, ACM Digital, BASE, EBSCOhost, Scopus, or Web of Science for clinical trials using various combinations of words. All articles that met the selection criteria were assessed using the Cochrane Collaboration’s risk of bias tool. For data analysis, the inverse variance and mean difference statistical method was used with Review Manager 5.3 software for Windows. According to the conclusion of each study (qualitative evaluation), only one clinical trial had results in favor of a COX-2 inhibitor when compared to placebo, one clinical study informed that a COX-2 was better that an active control, four studies showed similar analgesic efficacy to active controls, and one clinical study informed the analgesic effect of one celecoxib-caffeine combination in comparison with celecoxib alone and placebo (*n* = 337). The COX-2 inhibitors showed a decrease in the rescue analgesic consumption (*n* = 138; I^2^ = 15%; mean difference = −0.31; 95%CIs = −0.6 to −0.01), and lower pain intensity at four hours (*n* = 178; I2 = 0%; mean difference = −2.25; 95%CIs = −2.94 to −1.55; *p* = 0.00001) when compared to active controls after periodontal surgery. In conclusion, the data indicate that COX-2 agents produce better pain relief in comparison to placebo and other drugs after periodontal surgery.

## 1. Introduction

Postoperative pain control has a key role in all surgical fields [1]. In this regard, several kinds of drugs are available [2]. The local anesthetics [3,4], the non-steroidal anti-inflammatory analgesics (NSAIDs) [5], and glucocorticosteroids [6] are the most used in periodontal surgery. In addition, several postoperative pain management strategies are possible to employ after periodontal surgery [7]: multimodal analgesia [7,8,9], pre-emptive analgesia [10,11], and local administration of drugs [12]. These strategies for pain control have been demonstrated to decrease the postoperative analgesic intake [13,14] and reduce the pain intensity [15,16] following oral surgery.

Postoperative pain management after periodontal surgery is mainly performed with NSAIDs [17]. There are two types of NSAIDs, those that non-selectively inhibit the cyclooxygenase-2 (COX-2) enzyme [18,19] and the drugs that selectively inhibit that enzyme [20,21]. This last kind of drug has been used in clinical trials to determine its efficacy for pain management after periodontal surgery [22,23,24,25,26,27,28,29,30], and has been compared with placebo, other NSAIDs, and glucocorticosteroids [22,23,24,25,26,27,28,29,30]. The aim of this systematic review and meta-analysis was to evaluate the analgesic effect of the selective COX-2 drugs in comparison to the placebo, and active controls for pain control after periodontal surgery.

## 2. Material and Methods

### 2.1. Study Design

This systematic review was carried out according to the PRISMA guidelines at the Instituto de Investigación en Ciencias Médicas of the Centro Universitario de los Altos of the Universidad of Guadalajara [31,32]. The research was registered in PROSPERO (protocol ID: CRD42022340584).

### 2.2. Selection Criteria (PICO) [33]

#### 2.2.1. Inclusion Criteria

Population: Parallel-groups, or crossover clinical trials comparing the analgesic efficacy of COX-2 drugs and placebo or an active control—any non-COX-2 drug used to control postoperative pain—in periodontal surgery.

Interventions: Administration of a COX-2 drug.

Control: Patients were given a placebo or active drug.

Outcome: Analgesic intake, number of patients needing rescue analgesic medication, pain intensity using the visual analog scale (VAS score), and adverse effects.

#### 2.2.2. Exclusion Criteria

Trials with more than a 20% loss of postoperative follow-up.

### 2.3. Electronic Search

The most important medical database—PubMed—was employed to search clinical trials using a COX-2 drug in comparison to a placebo or/and active control in periodontal surgery. In addition, Google Scholar, ACM Digital, BASE, EBSCOhost, Scopus, or Web of Science were used for this purpose. The next COX-2 drugs were considered: “Celecoxib”, “etoricoxib”, “lumiracoxib”, “parecoxib”, and “valdecoxib”. Moreover, the next controls were used in the search: “Placebo”, “nonsteroidal anti-inflammatory analgesics”, “NSAIDs” “acetaminophen”, “diclofenac”, “ketorolac”, “ibuprofen”, “ketoprofen”, “glucocorticosteroids”, “methylprednisolone”, and “dexamethasone”. These words were employed with the next words to search clinical trials: “open-flap debridement”, “crown lengthening”, “mucoperiosteal flap surgeries for scaling and root planning”, and “periodontal surgery”. In PubMed, we defined the next filters: Article types (“Clinical trial”, “Controlled clinical trial”, and “Clinical study”), and Language (“English” and, “Spanish”). All clinical trials published up to February 2023 were considered for this study.

### 2.4. Assessment of Bias

The original Cochrane Collaboration’s risk of bias tool was used to assess the risk of bias of each clinical trial included [34,35]. The clinical trials with a high risk of bias (a red circle) were excluded from the qualitative and quantitative assessment [34,35]. This stage included the participation of two blinded independent researchers [36,37,38]. The studies with a high risk of bias (at least one red ball) as result of the original Cochrane Collaboration’s risk of bias tool.

### 2.5. Data Extraction

The first author, design study, treatment groups, size sample (*n*), dose, analgesic intake (means, SD, and, *n*), number of patients needing rescue analgesic medication, pain intensity using the VAS at 2, 4, 6, 8, 10, 12, and 24 postoperative hours (means, SD and, *n*), and adverse effects were obtained from each study.

The study by Steffens et al., 2011 was included in the statistical analysis as Steffens et al., 2011 to represent the data of celecoxib group, while Steffens et al., 2011 was used to include the data of etoricoxib group. For this reason and in order not to duplicate the *n* of the control group, the sample size was divided in half and placed in each comparison made with the COX-2 drugs.

### 2.6. Statistical Analysis

The inverse variance statistical method, and mean difference were used for data analysis with the Review Manager Software 5.3 for Windows. The fixed-effect model was employed to compare the analgesic intake while the pain intensity VAS scores were analyzed with the random-effect model. A global test with a *p*-value lower than 0.05 of the mean difference within the 95% confidence intervals (95% CIs) was regarded as statistically significant. The analysis of the data sensitivity was carried out to determine the influence of the weight of each study on the *p*-values [34,35,36,37,38,39].

## 3. Results

### 3.1. Searching and Evaluation of Bias

**Figure 1:**
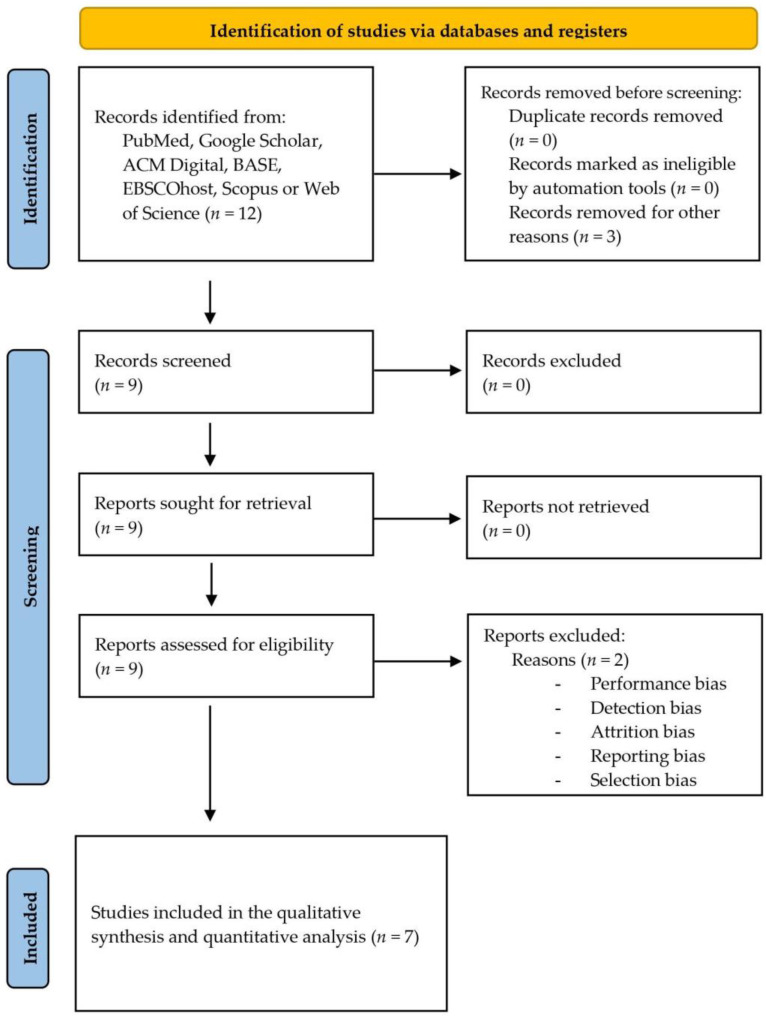
Study flow diagram.

A total of 12 articles were found by 2 independent researchers. Thereafter, the abstracts were read, and three reports were deleted because were not clinical trials. Next, 9 clinical trials were fully evaluated, and only 7 of these 9 clinical trials met the inclusion criteria for this systematic review [22,23,24,25,26,27,28,29,30] (Figure 1).

**Figure 2:**
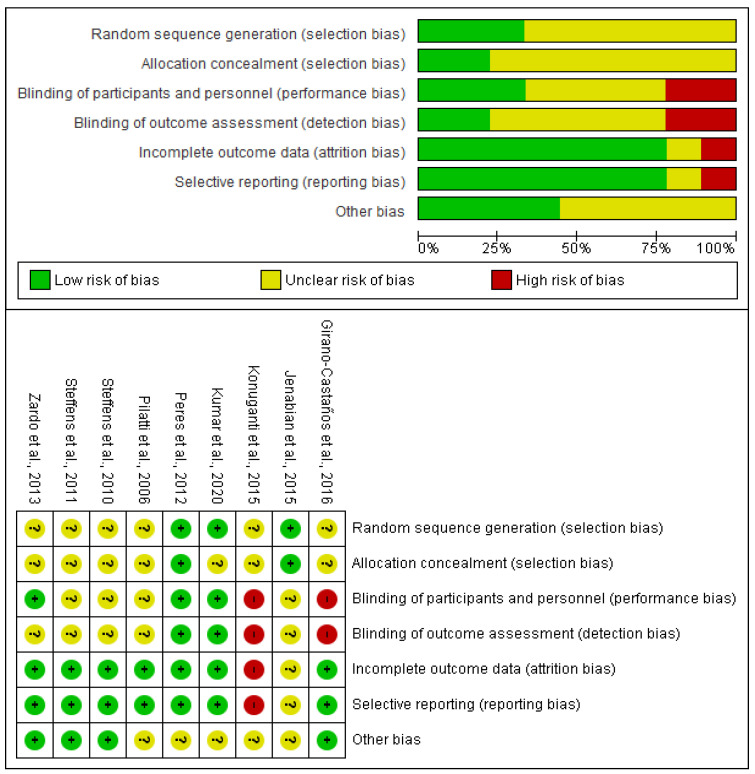
Evaluation of the risk of bias [22,23,24,25,26,27,28,29,30].

The assessment of bias shows that 7 out of 9 included articles in this systematic review present no high risk of bias [24,25,26,27,28,29,30]. The main problems of those 2 excluded clinical trials were the performance bias, detection bias, attrition bias, reporting bias, and selection bias [22,23] (Figure 2).

### 3.2. Qualitative Assessment

The qualitative analysis of the analgesic efficacy of COX-2 inhibitors was completed with 7 clinical trials (*n* = 337) [24,25,26,27,28,29,30]. The most widely used COX-2 inhibitor was Celecoxib 200 mg, which was used in 4 of the 7 clinical trials [24,26,28,30], followed by etoricoxib 90 mg [29] and 120 mg [27], and finally, lumiracoxib in a double-blind randomized clinical trial [25]. The main active control was dexamethasone, which was used in four clinical trials [25,26,27,29]. The doses of this drug used were 4 mg [25,26] and 8 mg [27,29]. According to the conclusion of each study, only one clinical trial had results in favor of a COX-2 inhibitor when compared to a placebo [28], one clinical study informed that a COX-2 was better than an active control [24], four studies showed similar analgesic efficacy to active controls [25,26,27,29], and one clinical study informed the analgesic effect of one celecoxib-caffeine combination in comparison with celecoxib alone and placebo [30]. For more details, such as ID study, first author, publishing year, treatments, sample size, details of patients, dental procedure, and evaluation, see Table 1.

### 3.3. Quantitative Evaluation

**Figure 3:**
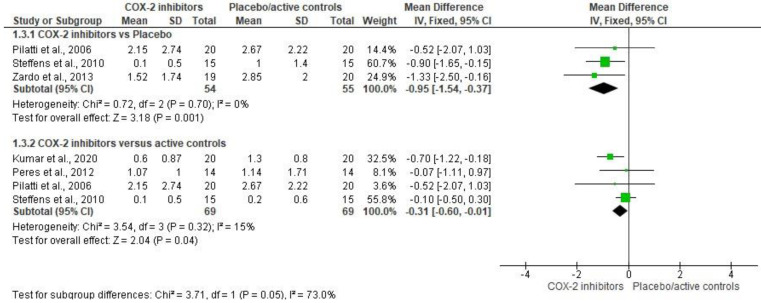
Assessment of rescue analgesic intake (*p* < 0.05) [24,25,26,27,29].

The evaluation of rescue analgesic intake in the postoperative period was made with 5 clinical trials (*n* = 227) [24,25,26,27,29]. The COX-2 inhibitors showed a decreasing of the rescue analgesic consumption when compared to placebo (*n* = 109; I^2^ = 0%; mean difference = −0.95; 95%CIs = −1.54 to −0.37; *p* = 0.001) [26,27,29] or active controls (*n* = 138; I^2^ = 15%; mean difference = −0.31; 95%CIs = −0.6 to −0.01; *p* = 0.04) [24,25,26,27,29] after periodontal surgery (Figure 3).

**Figure 4:**
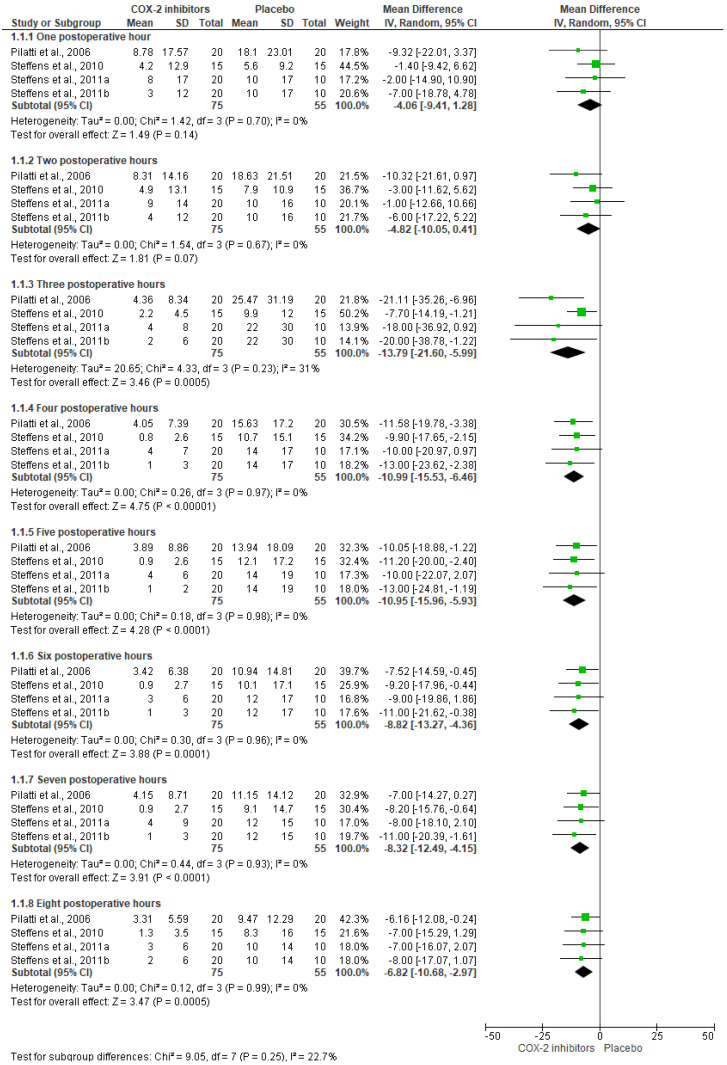
Postoperative pain intensity using COX-2 agents versus placebo in periodontal surgery (*p* < 0.05) [26,27,28].

The evaluation of the analgesic effectiveness of the COX-2 drugs was made with 6 clinical trials (*n* = 308) [24,25,26,27,28,30]. The pain control using COX-2 drugs in comparison with placebo was evaluated using 3 clinical trials (*n* = 130) [26,27,28]. Pooled analysis shows that patients who received the COX-2 inhibitors had lower pain scores when compared to placebo at 3 (*n* = 130; I^2^ = 31%; mean difference = −13.79; 95%CIs = −21.60 to −5.99; *p* = 0.0005; Figure 4), 4 (*n* = 130; I^2^ = 0%; mean difference = −10.99; 95%CIs = −15.53 to −6.46; *p* = 0.00001; Figure 4), 5 (*n* = 130; I^2^ = 0%; mean difference = −10.95; 95%CIs = −15.96 to −5.93; *p* = 0.0001; Figure 4), 6 (*n* = 130; I^2^ = 0%; mean difference = −8.82; 95%CIs = −13.27 to −4.36; *p* = 0.0001; Figure 4), 7 (*n* = 130; I^2^ = 0; mean difference = −8.32; 95%CIs = −12.49 to −4.15; *p* = 0.0001; Figure 4), and 8 postoperative hours (*n* = 130; I^2^ = 0%; mean difference = −6.82; 95%CIs = −10.68 to −2.97; *p* = 0.0005; Figure 4) [26,27,28].

**Figure 5:**
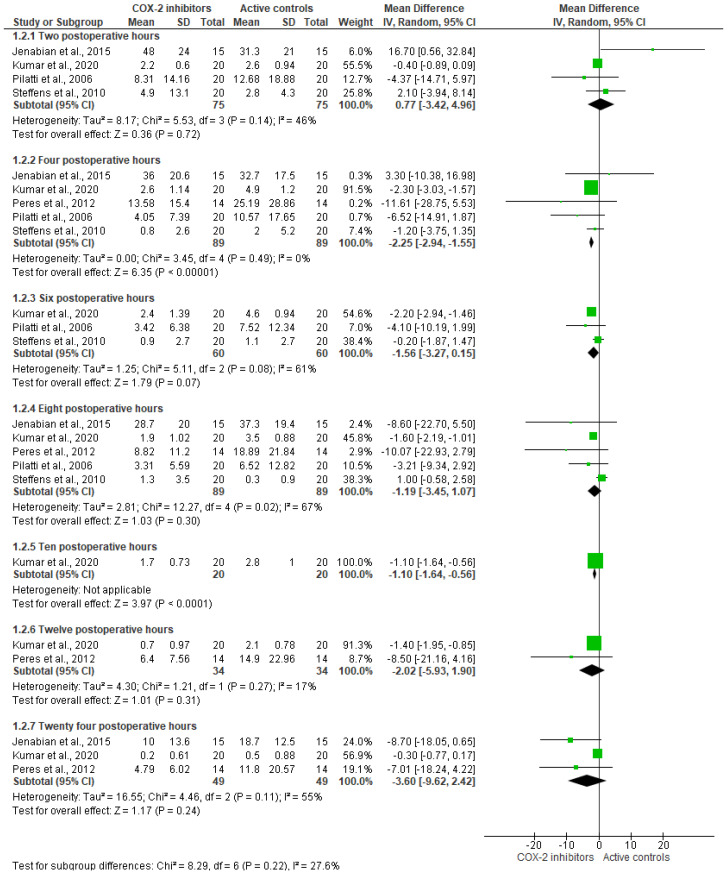
Postoperative pain control using COX-2 agents versus active controls in periodontal surgery (*p* < 0.05) [24,25,26,27,30].

Likewise, the assessment of the analgesic efficacy of COX-2 inhibitors in comparison to an active control was completed with 5 clinical trials (*n* = 178) [24,25,26,27,30]. In this sense, patients who were given COX-2 agents had minor pain intensity in comparison to those who were given active controls at 4 postsurgical hours only (*n* = 178; I^2^ = 0%; mean difference = −2.25; 95%CIs = −2.94 to −1.55; *p* = 0.00001; Figure 5) [24,25,26,27,30]. This was the only time when a statistical difference was detected. However, it is important to note that the trend of the data in this meta-analysis appears to be in favor of selective COX-2 enzyme inhibitors when visually compared to other NSAIDs or traditional glucocorticoids during the first 24 postoperative hours.

### 3.4. Sensitivity Analysis

Important changes in *p*-values were observed during the sensitivity analysis of analgesic consumption when the data from Kumar et al., 2020 were excluded [24]. The sensitivity analysis of the statistical analysis of the comparison of the COX-2 enzyme inhibitors compared to a placebo showed that the statistical differences were preserved despite the fact that the highest weight studies were excluded from the analysis. However, the statistical difference observed in the meta-analysis to compare the control of the postoperative pain of the COX-2 versus active controls was reversed when the data from the article by Kumar et al., 2020 [24], were removed from the analysis.

## 4. Discussion

To our knowledge, this is the first systematic review to use statistical analysis to pool data from double-blind randomized clinical trials that evaluated the efficacy of COX-2 drugs compared with placebo and active controls in patients with some type of periodontal surgery. Data from seven clinical trials [24,25,26,27,28,29,30], which had no bias according to the original Cochrane Collaboration’s risk of bias tool were employed to do the qualitative and quantitative comparison of the analgesic efficacy of the COX-2 drugs and active controls following periodontal surgery. The qualitative evaluation of this systematic review was made considering the information presented by the authors at the conclusion of the study. Thus, the analgesic efficacy of COX-2 enzyme selective agents shows that only one clinical trial reported results in favor of a COX-2 inhibitor compared to a placebo [28], and one clinical study reported that a drug COX-2 was better than an active control [24], four studies showed analgesic efficacy similar to active controls [25,26,27,29] and one trial reported the analgesic effect of a combination of celecoxib and caffeine compared with celecoxib alone and placebo [30]. On the other hand, the statistical analysis shows that the selective COX-2 inhibitors decreased the analgesic intake and postoperative pain when compared to placebo and other drugs after periodontal surgical treatment [24,25,26,27,28,29,30]. On the other hand, the main problems of the excluded clinical trials were the blinding of the research team, the patients, and the outcome evaluator. In addition, a clinical trial presented incomplete result data, and another one selectively reported. Table 2 shows the characteristics of the studies that were excluded from this systematic review and meta-analysis [22,23].

Based on the results of the qualitative and quantitative analysis of this report, and the consistency of the results, we could say that there is evidence on the analgesic efficacy of the COX-2 selective drugs (celecoxib [24,26,28,30], etoricoxib [27,28,29], and lumiracoxib [25]) in periodontal surgery. Moreover, a disadvantage of our systematic review and meta-analysis is that studies with different types of surgery were included, as indicated by the electronic search—open-flap debridement [24,27,28], crown lengthening [25,30], mucoperiosteal flap surgeries for scaling and root planning [26], and periodontal surgery [29]. In this sense, more studies with a low probability of bias, which provide more data on the analgesic efficacy of different doses of these drugs, are necessary. This could offer the possibility of individual evaluation of each COX-2 selective drug in the different kinds of periodontal surgery. It would even be interesting to establish a single type of periodontal surgery for the evaluation of analgesics, similar to mandibular third molar surgery. Currently, this surgical procedure is recognized by the FDA as a 24 h characterized acute pain model, in which the postoperative pain peaks that will occur in the patient are known, allowing the clinical evaluation of existing drugs and the development of new molecules with antalgic properties [7,19].

After periodontal surgery, there are different complications that patients can experience [40,41]. Undoubtedly, the main complication after periodontal surgery is postoperative inflammatory pain that occurs in the immediate outpatient period, within the first 24 to 72 h [41], for which the dentist, specialist in periodontics, or oral surgeon can use different types of drugs, mainly NSAIDs [42], without being limited to these, depending on the intensity of the pain, they could make complementary use of other types of drugs such as glucocorticoids, e.g., dexamethasone [23]. Additionally, there are two different approaches to the treatment of postoperative inflammatory pain to consider. The first, preventive analgesia, is performed when a drug is administered to the patient before the surgical wound occurs [7]. Second, and no less important, is multimodal analgesia, which consists of using combinations of drugs before (preventive analgesia) or after a surgical event [43,44,45]. The combination used is based on the fact that the drugs must have different mechanisms of action, different types of onsets of the analgesic effect, and different duration of the analgesic effect. In this way, pain is treated by blocking different chemical mediators at different levels of the central nervous system [44,45]. Finally, when the preventive analgesia strategy is used with a single drug or under a multimodal approach, the maximum plasma concentration must be taken into account, so that the drug has its greatest presence in the blood and can reach the target immediately in the bed. surgical [46,47,48,49,50,51,52,53,54,55,56]. In the case of multimodal analgesia using a combination of drugs, it must be taken into account that at least one of the drugs used is at its maximum plasma concentration at the time of incision [46,47,48,49,50,51,52,53,54,55,56].

In this systematic review and meta-analysis, it was not possible to assess the adverse effects of COX-2 drugs because no studies reported adverse effects. However, some meta-analyses have evaluated the adverse reactions of COX-2 agents in comparison with placebo and against other types of drugs. A meta-analysis evaluated the adverse events of COX-2 drugs compared to ibuprofen. The authors found that patients receiving celecoxib had fewer adverse reactions (nausea and vomiting) compared with the ibuprofen group after a third molar surgery [57]. Similar results were reported by Isiordia-Espinoza et al., 2022 on the safety profile of celecoxib in comparison to active controls following third molar surgery [35]. Another meta-analysis found no difference in adverse events (nausea, vomiting, dizziness, headache, and alveolar osteitis) between etoricoxib and traditional NSAIDs in a third molar surgery [58]. A similar risk of adverse effects has been reported between celecoxib and traditional NSAIDs with respect to gastrointestinal and vascular effects and acute myocardial infarction. On the other hand, the risk of renal or cardiovascular disease due to these same agents has been ruled out [35]. On the other hand, a limited increase in adverse effects has been reported when comparing etoricoxib with a placebo. However, the risk of gastrointestinal adverse effects was higher with diclofenac than with etoricoxib in patients with osteoarthritis. Finally, there is no evidence of serious adverse effects with the use of etoricoxib [58]. Our research team recommends increasing the number of clinical trials that test COX-2 selective agents in periodontal surgery to confirm their analgesic and anti-inflammatory efficacy, also considering the reporting of adverse effects essential, which will allow us to determine if there is a larger susceptible population.

Some of the advantages of this systematic review and meta-analysis were that only double-blind randomized clinical trials that scored high in quality according to the Cochrane tool were included [34,35,36,37,38], and rigorous statistical analysis, such as in the case of the use of fixed and random effects models, the values of heterogeneity were considered for this purpose. When the inconsistency was between 0% and 30% (according to the value of I^2^), the fixed effects model was used, while when the value of I^2^ was greater than 30%, the random effects model was used, which is more conservative [36,59]. Another advantage of this study was the number of databases in the area of health sciences consulted—PubMed, Google Scholar, ACM Digital, BASE, EBSCOhost, Scopus, and Web of Science —which allowed us to detect scientific articles of interest. This systematic review and meta-analysis has several disadvantages, i.e., few clinical trials comparing the intervention of interest were detected, and their methodology could be better, mainly in relation to the measurement of endpoint variables, which provide an estimate of analgesic efficacy [34,35,60]. Regarding the above, the evaluation of patient satisfaction according to the treatment received could be included [61]. These clinical trials had small sample sizes, and the analysis was completed by combining data from different COX-2 inhibitors in the same treatment group [34,35,54,62]. It was not possible to carry out an individual evaluation of the analgesic efficacy of each one of the COX-2 inhibitors, the best pharmaceutical presentation—solid, semi-solid, or liquid forms—and determine the best route of administration [34,35,62].

Meta-analyses are tools that integrate the results of studies that met a certain methodological and scientific rigor to answer a research question that still requires an answer. That is, these studies, despite having been carried out with adequate scientific rigor, present inconsistent or even contradictory results, which justify the use of statistical analysis as a whole, and thus draws conclusions based on all the evidence. available. However, there is a big problem here, publication bias. The probability that a scientific article will be published will always be higher when it presents positive results—a statistical difference in its results—compared to when negative results or no statistical difference are reported. For this reason, many meta-analyses could present this disadvantage. The foregoing has the consequence of reaching incorrect conclusions. Some tools used to detect publication bias are selection models (usually employed as sensitivity analyses), funnel plots (usually plotting the inverse of standard errors), Begg’s rank test, and Egger’s regression test. However, despite the above, detecting or combating publication bias of a meta-analysis is very difficult [36,37,61,63,64]. In this study, the observed statistical differences in rescue analgesic intake and pain management at 4 h postoperatively between the comparison of COX-2 inhibitors and active controls were lost.

## 5. Conclusions

Data indicate that COX-2 agents produce better pain relief in comparison to placebo and other drugs after periodontal surgery. Our research group recommends increasing the number of double-blind randomized clinical trials evaluating the analgesic efficacy and safety of COX-2 agents in comparison with other NSAIDs or glucocorticoids after some type of periodontal surgery. This would allow us to establish clinical guidelines for the use of these drugs in periodontal surgery, according to their analgesic efficacy and adverse effects.

## Figures and Tables

**Table 1:** Characteristics of the studies that met the selection criteria.

ID Study and Study Design	Treatments (*n*)	Details of Patients, Dental Procedure, and Evaluation	Important Results (Conclusions)
Kumar et al., 2020 [24] Randomized, double-blind, parallel clinical trial. Single-dose study. Preemptive analgesia.	Group A: Celecoxib 200 mg (*n* = 20). Group B: Acetaminophen 500 mg (*n* = 20). Drugs were given by oral route.	Healthy patients aged between 20 years to 50 years were included. Patients underwent open-flap debridement. The inferior alveolar nerve block was made with 2% lidocaine, and local infiltration with 4% articaine with 1:100,000 epinephrine. The rescue analgesic was not informed. The number of patients taking rescue analgesic medication, the time to first analgesic medication after surgery, and the pain intensity were recorded.	Celecoxib was more effective than acetaminophen for control of pain after periodontal surgery.
Peres et al., 2012 [25] Randomized, double-blind, parallel clinical trial. Single-dose study. Preemptive analgesia.	Group A: Lumiracoxib 400 mg (*n* = 14). Group B: Dexamethasone 4 mg (*n* = 14). Drugs were administered orally.	Patients needing to be submitted to periodontal surgery for crown lengthening were selected. Local infiltration was made using 2% lidocaine and 1:100,000 epinephrine. Dipyrone 500 mg rescued analgesic medication was employed. The anxiety level, the number of pills of dipyrone after surgery, edema, and pain intensity were assessed.	Lumiracoxib and dexamethasone had similar anti-inflammatory and analgesic effects.
Pilatti et al., 2006 [26] Randomized, triple-blind, crossover, clinical assay. Multiple-dose study. Preemptive analgesia.	Group A: Celecoxib 200 mg (*n* = 20). Group B: Dexamethasone 4 mg (*n* = 20). Group C: Placebo (*n* = 20). Drugs were given by oral route 1 h before surgery and placebo and dexamethasone were given 8 h after of first dose and celecoxib 12 h after of the first dose.	Patients aged 27 years and 52 years were included. Patients underwent mucoperiosteal flap surgery, scaling, and root planing on at least three quadrants were included. Local anesthesia was carried out with 2% lidocaine and 1:100,000 epinephrine. Acetaminophen 750 mg rescue analgesic medication was used. The stress, anxiety, and pain intensity were evaluated.	Celecoxib was better when compared to placebo. Dexamethasone was not more effective than a placebo. The authors concluded that celecoxib and dexamethasone were effective for pain control after periodontal surgery.
Steffens et al., 2010 [27] Randomized, triple-blind, crossover, clinical assay. Single-dose study. Preemptive analgesia.	Group A: Etoricoxib 120 mg (*n* = 15). Group B: Dexamethasone 8 mg (*n* = 15). Group C: Placebo (*n* = 15). Drugs were administered by oral route.	Patients aged 18 years to 56 years and needing open-flap debridement surgery were selected. Local anesthesia was given using 2% mepivacaine and 1:100,000 epinephrine. Acetaminophen 750 mg rescue analgesic medication was used. The stress, anxiety, total analgesic intake, and pain intensity were evaluated.	Both etoricoxib and dexamethasone are effective for pain control after periodontal surgery.
Steffens et al., 2011 [28] Randomized, double-blind, parallel clinical assay. Multiple-dose study. Preemptive analgesia.	Group A: Celecoxib 200 mg 1 h before surgery and another 200 mg dose 12 h after the first dose (*n* = 20). Group B: Etoricoxib 120 mg 1 h before surgery (*n* = 20). Group 3. Placebo 1 h before surgery (*n* = 20).	Patients aged 18 years to 56 years old with needing a mucoperiosteal flap. The anesthesia was completed using 2% mepivacaine with 1:100,000 epinephrine. Paracetamol 750 mg rescue analgesic medication was employed. Pain intensity by VAS, stress, anxiety, and analgesic intake were evaluated.	Similar analgesic effectiveness between celecoxib and etoricoxib was observed. Both drugs were better than the placebo.
Zardo et al., 2013 [29] Randomized, double-blind, parallel clinical trial. Single-dose study. Preemptive analgesia.	Group A: Etoricoxib 90 mg (*n* = 19). Group B: Dexamethasone 8 mg (*n* = 19). Group C: Placebo (*n* = 20). Drugs were given by oral route 1 h before surgery.	Patients aged 19 years to 67 years with indications of periodontal surgery were included. Anesthesia was made using 2% lidocaine—1:100,000 epinephrine. Paracetamol 750 mg rescue analgesic medication was utilized. Rescue analgesic intake, pain intensity, and adverse effects were measured.	Etoricoxib and dexamethasone are effective for the control of pain in periodontal surgery.
Jenabian et al., 2015 [30] Randomized, double-blind, parallel clinical trial. Multiple-dose study. Preemptive and postoperative analgesia.	Group A: Ibuprofen 400 mg (*n* = 15). Group B: Celecoxib 200 mg (*n* = 15). Group C: Celecoxib 200 mg plus caffeine 30 mg (*n* = 15). Drugs were given an hour before surgery and 1 h, 8 h, 16 h, and 24 h after crown lengthening surgery.	Patients aged 20 years to 60 years who needed crown lengthening surgery were included in this study. The local anesthetic used in the surgical procedures was not reported. Paracetamol-codeine rescue analgesia was used. Pain was evaluated by VAS	The celecoxib plus caffeine combination showed better analgesic efficacy when compared to other treatments in crown lengthening surgery.

**Table 2:** Characteristics of the excluded studies.

ID Study and Study Design	Treatments (*n*)	Details of Patients, Dental Procedure, and Evaluation	Important Results (Conclusions)
Girano-Castaños et al., 2016 [22] Randomized, parallel clinical trial. Single-dose study. Preemptive analgesia.	Group A: Etoricoxib 120 mg (*n* = 15). Group B: Ketorolaco 10 mg (*n* = 15). Drugs were given by oral route 1 h prior surgery.	Patients who required periodontal plastic surgery due to periodontal recession were included. The anesthetic technique and the medication used for this purpose were not specified. Paracetamol 500 mg rescue analgesic was used. Pain intensity using the VAS and a verbal scale was recorded.	Similar analgesic efficacy between etoricoxib and ketorolac after periodontal surgery was observed. No adverse effects were reported.
Konuganti et al., 2015 [23] Randomized, parallel clinical trial. Single-dose study. Preemptive analgesia.	Group A: Etoricoxib 120 mg (*n* = 20). Group B: Dexamethasone 8 mg (*n* = 20). Group C: Placebo (*n* = 20). Drugs were administered orally 1 h before surgery.	Patients aged 18 years to 56 years who needing open flap debridement surgery. The anesthetic technique and the medication used for this purpose were not specified. Paracetamol 650 mg rescue analgesic was used. The the number of pills after surgery, and pain intensity were assessed.	Similar analgesic effectiveness between etoricoxib and dexamethasone was observed. No adverse effects were reported.

## Data Availability

Data is contained within the article.
